# Dysregulation of tristetraprolin and human antigen R promotes gastric cancer progressions partly by upregulation of the high-mobility group box 1

**DOI:** 10.1038/s41598-018-25443-3

**Published:** 2018-05-04

**Authors:** Hao Wang, Yigang Chen, Jian Guo, Ting Shan, Kaiyuan Deng, Jialin Chen, Liping Cai, Hong Zhou, Qin Zhao, Shimao Jin, Jiazeng Xia

**Affiliations:** Department of General Surgery and Translational Medicine Center, Nanjing Medical University Affiliated Wuxi Second Hospital, Wuxi, 214002 China

## Abstract

Aberrant expression of ARE-binding proteins (ARE-BPs) plays an important role in several diseases, including cancer. Both tristetraprolin (TTP) and human antigen R (HuR) are important ARE-BPs and always play opposite roles in regulating target mRNAs. Our previous work has demonstrated that TTP expression is decreased in gastric cancer (GC). In this study, we reported that HuR was elevated in GC cell lines and gastric cancer patients and that decreased TTP expression partly contributed to the elevated HuR levels by regulating its mRNA turnover. We also observed that dysregulation of TTP and HuR elevated the high-mobility group box 1 (HMGB1) expression in different ways. HuR promoted HMGB1 expression at translational level, while TTP regulated HMGB1 mRNA turnover by destabilizing its mRNA. Increased HuR promoted cancer cell proliferation and the metastasis potential partly by HMGB1. Using immunohistochemistry, we observed that both positive cytoplasmic and high-expression of nuclear HuR were associated with poor pathologic features and survival of GC patients. In conclusion, this study demonstrated that dysregulation of the TTP and HuR plays an important role in GC. Moreover, high HuR nuclear expression or aberrant cytoplasmic distribution may serve as a predictor of poor survival.

## Introduction

Gastric cancer has been estimated as the fifth most common neoplasm and third leading cause of cancer deaths worldwide. However, almost half of these cases occur in China and most of them are diagnosed at an advanced stage^1^. In 2012, the crude GC incidence in China was 42.93/100000 in males and 19.03/100000 in females. Furthermore, the crude mortality of GC was 29.67/100000 in males and 14.02/100000 in females^2^. Therefore, it is urgent and vital to determine effective biomarkers or therapeutic targets for treating GC.

Adenosine-uridine (AU)-rich elements (AREs), located in the 3′ untranslated regions (UTR) of gene transcripts, are important cis-acting factors and mediate complex posttranscriptional regulations. Approximately 7% of protein-coding gene transcripts are estimated to have AREs in their 3′ UTR^3^. ARE-binding proteins (ARE-BPs), such as tristetraprolin (TTP), human antigen R (HuR), and AU-binding factor 1 (AUF1), interact with target ARE-containing mRNAs and regulate the stability or translation of these transcripts. This post-transcriptional regulation has been demonstrated to play an important role in carcinogenesis^4,5^.

TTP (also known as ZFP36, TIS11) is a member of the TIS11 family and contains two repeat CCCH zinc fingers domains, which mediate the interaction between TTP and target transcripts^3^. Decreased expression of TTP has been observed in various types of cancers, such as gastric, pancreatic, breast, colon and lung cancers, among others^3,6,7^. Generally, TTP interacts with mRNAs and destabilizes target transcripts by recruiting deadenylase complexes^6^. TTP acts as a tumor suppressor by regulating several key factors that have been implicated in several aspects of tumor progression^3,6^. Conversely, some ARE-BPs, such as HuR and polyadenylate-binding protein-interacting protein 2 (PAIP2), act as stabilizing factors for ARE-containing mRNAs. HuR (also known as ELAVL1) is a typical mRNA-stabilizing factor and belongs to the embryonic lethal abnormal vision (ELAV)-like family. Elevated expression or aberrant nuclear/cytoplasmic distribution of HuR has been observed in several types of cancers and can promote tumorigenesis by stabilizing tumor-promoting transcripts^8,9^. Apart from its role of stabilizing mRNAs, HuR may also promote the translation of several target mRNAs^10^.

Both TTP and HuR are important ARE-BPs, and their dysregulation plays an important role in cancer^5,11^. The relationship between TTP and HuR is complex and interesting. TTP can downregulate the mRNA of HuR^11,12^. Moreover, thousands of overlapping binding sites of TTP and HuR have been found in more than 1000 genes^13^. Furthermore, both of TTP and HuR are highly regulated by posttranscriptional modifications in cells. For example, phosphorylation of different residues of TTP and HuR plays an essential role in regulating their expression, function and localization^5,14,15^. Dysregulation of TTP and HuR has been demonstrated to promote the progression of cancers by modulating several downstream targets^3^.

Our previous work has demonstrated that TTP is downregulated in GC and acts as a tumor suppressor by down-regulating IL-33 expression. Decreased TTP expression is associated with poor clinical features and survival^16^. In this study, we aimed to investigate whether HuR was elevated or showed aberrant nuclear/cytoplasmic translocation in GC. We also explored the relationship between HuR and TTP in GC.

The high-mobility group box 1 (HMGB1), which was first identified as a highly conserved non-histone chromosomal protein, is extensively expressed in all eukaryotic cells^17,18^. HMGB1 is a multifunctional factor based on its location. Intracellular HMGB1 acts as a chromosome sustainer, DNA chaperone, autophagy regulator, inhibitor of apoptosis, and so on. Moreover, HMGB1 is a prototypical damage-associated molecular pattern molecule (DAMP). HMGB1 can be released into the extracellular space in two ways: it can be actively secreted by immune cells or neuronal cells as well as passively released by injured, dying or dead cells. Extracellular HMGB1 can bind to various signal transduction cell receptors and activate downstream signaling pathways. The intracellular and extracellular functions of HMGB1 enable it to play a significant role in inflammation, immunity and cancer^19–21^. Overexpression and increased serum levels of HMGB1 have been observed in several types of cancers, including GC^18,19^. The role of elevated HMGB1 levels in cancers is paradoxical because current research has demonstrated that HMGB1 has both tumor-promoting and suppressing effects in cancer progression. However, the mechanism by which HMGB1 expression is elevated in cancers is not clear^20,21^.

In this study, we observed that the expression of HuR was evidently elevated in cell lines and GC patients. Decreased TTP expression contributed to the increased HuR level by regulating its mRNA turnover. Moreover, dysregulation of the TTP and HuR contributed to increased HMGB1 expression in two different ways. HuR promoted HMGB1 expression at translational level, while TTP regulated HMGB1 mRNA turnover by destabilizing its mRNA. Increased expression of HuR in a xenograft tumor model in nude mice promoted the proliferation of GC cells. Using immunohistochemistry, we found that both positive cytoplasmic and highly expressed nuclear HuR were associated with poor pathological features and the survival of GC patients.

## Results

### The mRNA and protein expression of HuR is increased in GC cell lines and GC patients

**Figure 1:**
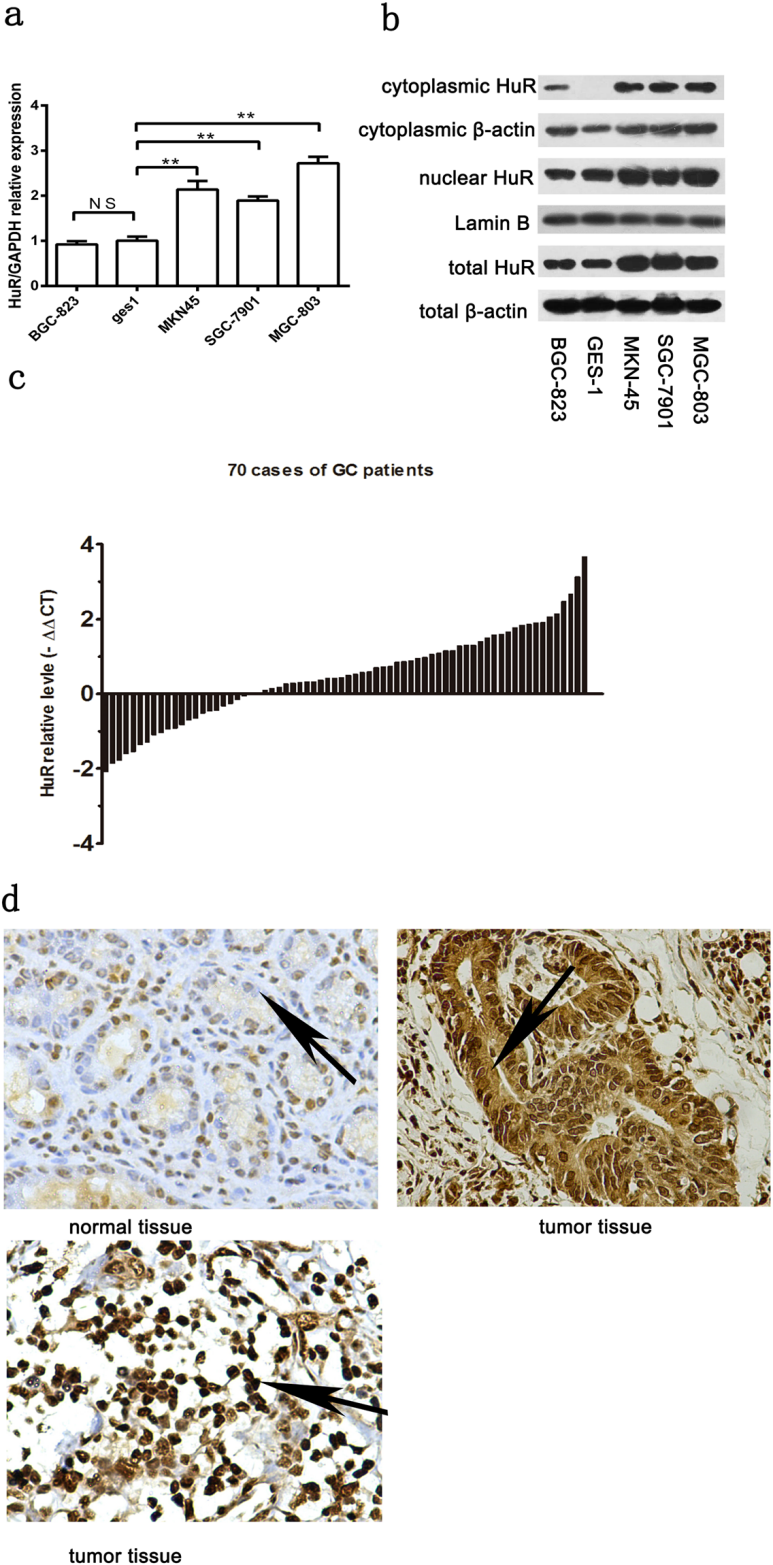
Expression of HuR was elevated in GC. (**a**) HuR mRNA expression was evidently higher in MGC-803, SGC-7901 and MKN-45 cell lines than in the non-malignant GES-1 cells. The data were represented as the mean ± SD. **p < 0.01. (**b**) Positive cytoplasmic HuR expression was detected in MGC-803, SGC-7901, MKN-45, and BGC-823 cells, whereas no cytoplasmic expression of HuR was found in GES-1 cells. (**c**) Increased HuR mRNA levels were observed in 70% (49/70) patients. (**d**) Representative images of the immunohistochemistry analysis of HuR expression in tissues. Normal mucosae only showed a weak nuclear expression of part cells (top and left). However, cancer tissues exhibited a pattern of high nuclear expression (bottom and left), and an aberrant cytoplasmic distribution (top and right) was detected in (22/70) cases.

To understand whether HuR is highly expressed in GC, expression of the HuR mRNA and protein were examined in four GC cell lines (MGC-803, BGC-823, SGC-7901, MKN-45) and in non-malignant GES-1 cells by quantitative real-time PCR (qRT-PCR) and western blotting. We observed that HuR mRNA expression was evidently higher in the MGC-803, SGC-7901 and MKN-45 cell lines than GES-1. However, there was no significant difference in expression between BGC-823 and GES-1 cells (Fig. 1a). Because HuR is an important nuclear/cytoplasmic shuttling protein, we extracted the cytoplasmic and nuclear HuR proteins and examined them by western blotting. Positive cytoplasmic HuR protein expression was detected in MGC-803, SGC-7901, MKN-45, and BGC-823 cells, whereas no cytoplasmic expression of HuR was found in GES-1 cells (Fig. 1b). These results demonstrated that the mRNA and protein expression of HuR were evidently higher in GC cell lines.

To investigate whether HuR expression is higher in GC patients as well, mRNA expression of HuR was explored by qPCR in cancerous and correspondingly matched normal mucosa collected from 70 patients. Increased HuR mRNA levels were observed in 70% (49/70) patients (Fig. 1c). Subsequently, we used immunohistochemistry to investigate the HuR protein levels in these 70 patients (Fig. 1d). Compared to the HuR staining in adjacent normal tissues (mean immunohistochemistry score (IHS): 4.26 ± 1.39; IHS range, 1–8), HuR staining in cancerous tissues was significantly higher (mean IHS: 7.20 ± 1.81, IHS range, 3–12) (*P* < 0.001). Aberrant positive cytoplasmic HuR expression was also detected in 31.42% (22/70) of cancerous tissues.

### Tristetraprolin downregulates HuR expression in GC cells

**Figure 2:**
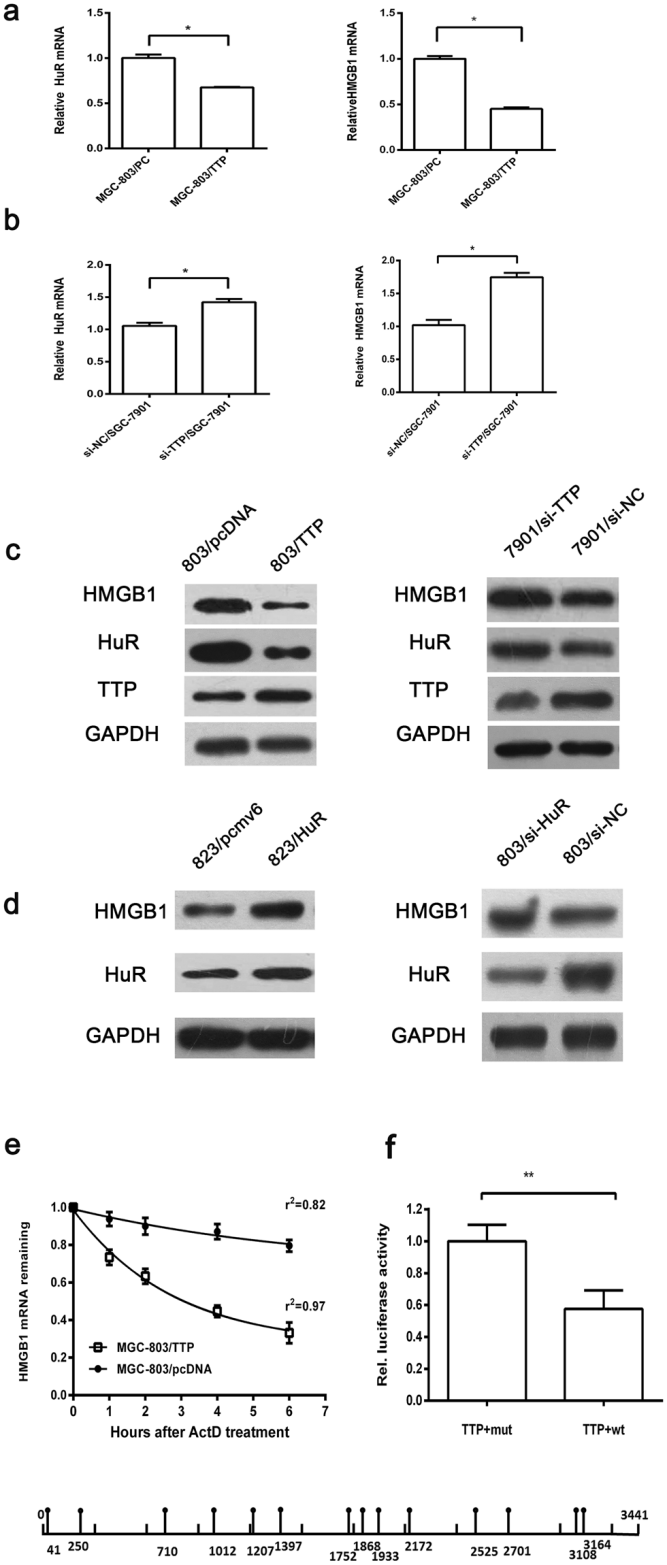
HuR was downregulated by TTP, and dysregulation of TTP and HuR regulated the expression of HMGB1 in different ways. (**a**–**c**) TTP regulated the expression of HuR and HMGB1 at the mRNA level. (**d**) HuR upregulated the expression of HMGB1 at the translational level. (**e**) The HMGB1 mRNA decay curve in actinomycin D-treated MGC-803/TTP and MGC-803/pcDNA cells. The half-life of HMGB1 mRNA in MGC-803/TTP cells was shorter than that in MGC-803/pcDNA cells after 6 hours of actinomycin D treatment. The data were represented as the mean ± SD, r^2^: goodness of fit, **p < 0.01. (**f**) Luciferase assay in 293 T cells revealed that AREs within the HMGB1 mRNA 3′UTR mediated the inhibitory of TTP. And the schematic showed the potential binding sites of TTP within the HMGB1 mRNA 3′ UTR. Results shown on the graph represent the mean ± S.D. of three independent experiments, **p < 0.01.

TTP and HuR are two important cancer-related ARE-BPs, and researchers have also demonstrated that TTP can downregulate the mRNA of HuR^12^. Previously we found that TTP is down-regulated in GC. To investigate whether TTP regulates the expression of HuR and whether a TTP deficiency contributes to the overexpression of HuR in GC, we transfected TTP expression plasmids (pcDNA-TTP) and the empty pcDNA3.1 control vectors into MGC-803 cells (relatively low expresser of TTP) and then examined HuR expression by qRT-PCR and western blotting. We observed that the HuR mRNA and protein expression were evidently decreased in MGC-803/TTP cells (Fig. 2a,c). Another report demonstrated that silencing of HuR induced an increase in TTP in human pulmonary microvascular endothelial cells^22^. We transfected pCMV6-HuR plasmids or pCMV6-Entry vectors into BGC-823 cells (relatively low expresser of HuR) and measured TTP expression. However, no changes in TTP mRNA or protein expression were detected (data not shown).

We further knocked down TTP expression in SGC-7901 cells (relatively high expresser of TTP) using TTP-siRNAs and examined the mRNA and protein expression of HuR, and the results confirmed the regulatory effect of TTP on HuR (Fig. 2b,c). Because TTP downregulates HuR mRNA levels, we analyzed the mRNA expression of HuR and TTP in 70 GC patients. Although the results of the Chi-squared test demonstrated no significant difference between TTP and HuR mRNA expression, we observed that 48.6% (34/70) of these GC patients displayed a pattern of high expression of HuR mRNA accompanied with decreased TTP mRNA levels (Table 1).

**Table 1:** The relationships between TTP and HuR, HMGB1 mRNAs were analyzed in 70 cases of GC patients.

Variable (mRNA)	TTP mRNA expression in cancer tissue					*P* value
	cases	decreased	%	increased	%	
HuR-increased	49	34	69.4	15	30.6	0.323
HuR-decreased	21	12	57.1	9	42.9	
HMGB1-increased	46	32	69.6	14	30.4	0.347
HMGB1-decreased	24	14	58.3	10	41.7	

### HuR and TTP regulate the expression of HMGB1 in different ways

Overexpression of HMGB1 in cancer cells and elevated serum levels in GC have been demonstrated in several studies^17,23,24^. Elevated HMGB1 expression is associated with poor prognosis in patients. However, the mechanisms by which HMGB1 is overexpressed in cancer were not clear^20^. It was reported that in the progression of myogenesis, HuR increased the expression of HMGB1 by suppressing the translational inhibition mediated by miR-1192^25^. To explore whether elevated HuR contributes to overexpression of HMGB1 in GC, we analyzed the mRNA and protein expression of HMGB1 after HuR had been overexpressed or knocked down in BGC-823 and MGC-803 cells. Expression of HMGB1 protein but not mRNA (data not shown) was affected by the changes in HuR expression, which indicated that elevated HuR expression in GC was a contributor to HMGB1 overexpression (Fig. 2d).

Next, we analyzed the 3′UTR of the HMGB1 mRNA and observed that it contained 14 potential binding sites (AUUUA) for TTP. Therefore, we explored whether TTP regulates HMGB1 expression. We observed that increased TTP significantly decreased the expression of HMGB1 at both the mRNA and protein levels, while knocking down TTP increased HMGB1 expression (Fig. 2a–c). To determine whether the decreased HMGB1 mRNA was the result of the regulation of its mRNA stability by TTP, we examined the half-life of HMGB1 mRNA in actinomycin D-treated MGC-803 cells transfected with TTP plasmids or the empty vectors. After 6 hours of actinomycin D treatment, approximately 33.2% of HMGB1 mRNA remained in MGC-803/TTP cells, whereas approximately 79.6% of HMGB1 mRNA was retained in the control cells (Fig. 2e). To further explore whether TTP could interact with 3′ UTR of HMGB1 mRNA directly, luciferase assay was performed using the wild and mutant types of HMGB1 ARE. The results of luciferase assay verified that TTP could regulate HMGB1 by binding to its ARE directly (Fig. 2f). Collectively, these results suggested that TTP destabilizes the mRNA of HMGB1 by binding to its ARE in GC cells. Next, we also detected the HMGB1 mRNA levels in the 70 paired cancer samples and analyzed their correlation with TTP mRNA. However, no significant relation was observed between HMGB1 and TTP expression at the mRNA level (Table 1). These results indicated that elevated HMGB1 in GC was partly due to the dysregulation of TTP and HuR, and that TTP regulated the expression of HMGB1 at the mRNA level by binding to its ARE, while elevated HuR promoted HMGB1 expression at translational level.

### Elevated HuR promotes GC cell proliferation partly mediated by HMGB1 *in vitro*

**Figure 3:**
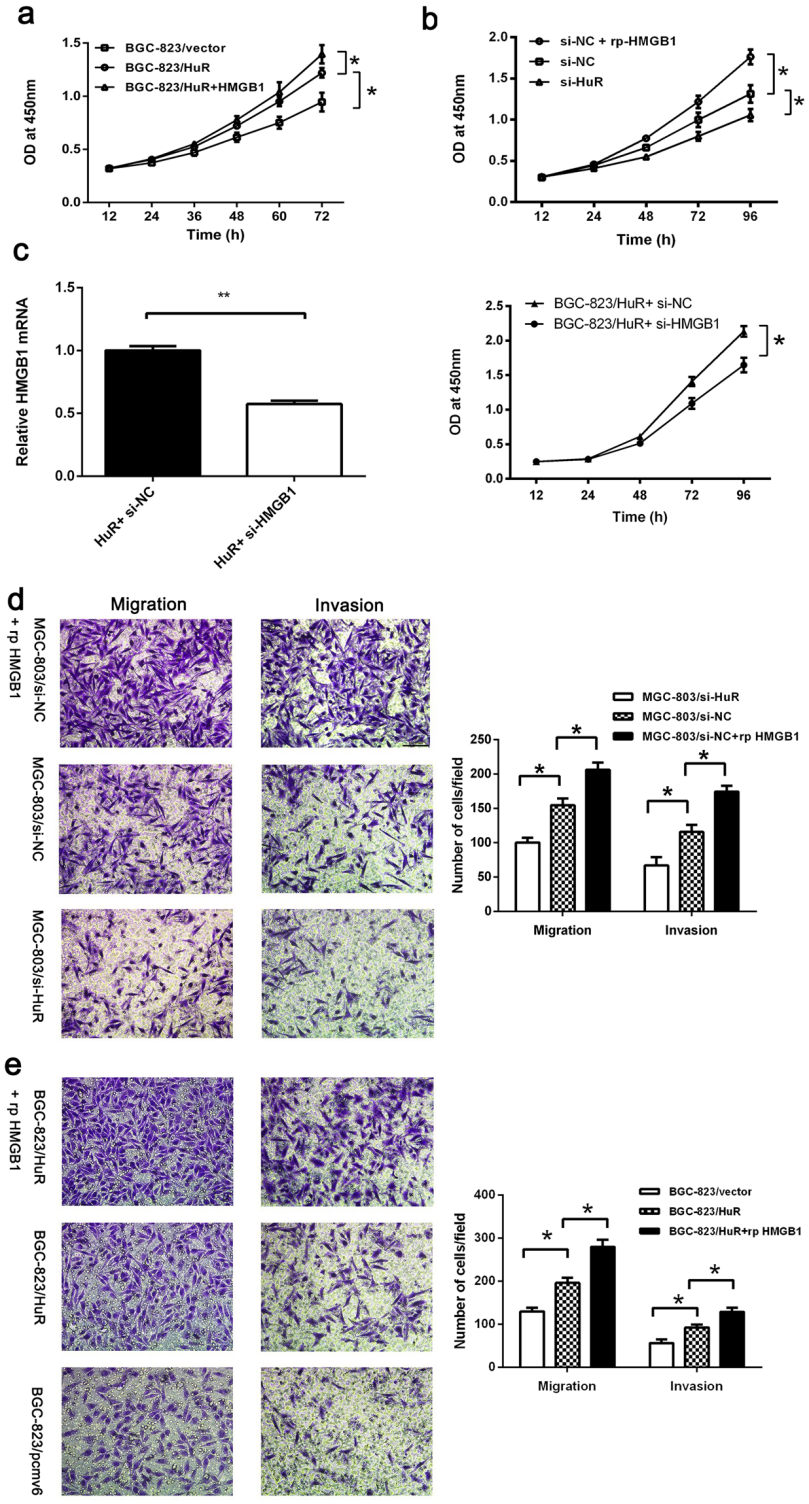
Elevated HuR promoted GC cell proliferation, migration and invasion partly mediated by HMGB1 *in vitro*. (**a**,**b**) Elevated HuR expression promoted GC cell proliferation while decreased expression of HuR inhibited cell proliferation. Additional rp-HMGB1 (100 ng/ml) promoted the proliferation of GC cells. (**c**) HuR plasmids and siRNA-HMGB1 (or siRNA-control) were cotransfected into BGC-823 cells and analyzed by CCK-8. Knockdown of HMGB1 inhibited the proliferation induced by HuR overexpression (right). Expressions of HMGB1 in cells were determined by qRT-PCR (left). (**d**,**e**) Elevated HuR levels promoted while decreased HuR levels inhibited the migration and invasion ability of cells partly by HMGB1. The data were represented as the mean ± SD. *p < 0.05, **p < 0.01.

HuR plays an important role in cancer cell proliferation by increasing the levels of proliferation related regulators (including cyclins, epidermal growth factor, c-Myc, eukaryotic translation initiation factor and others) and regulating several anti-apoptotic factors and signaling pathways^3^. To further explore whether elevated HuR expression influences the proliferation of GC cells, we transfected HuR plasmids and the control empty vectors into BGC-823 cells. Using the Cell Counting Kit-8 (CCK-8) assay, we observed that overexpression of HuR led to a significant increase in the proliferation of cancer cells (Fig. 3a). Next, we knocked down the expression of HuR in MGC-803 cells using siRNA-HuR and siRNA-control and analyzed by CCK-8. The results revealed that decreased HuR expression suppressed GC cell proliferation (Fig. 3b).

Because HMGB1 acts as a promoter of cell proliferation, we tested whether HuR-mediated acceleration of cell proliferation was correlated with extracellular HMGB1 levels. We treated BGC-823/HuR and MGC-803/siRNA-control cells with human recombinant HMGB1 (rp-HMGB1) (100 ng/ml) and analyzed them using the CCK-8 assay. The results revealed that the proliferation of GC cells treated with rp-HMGB1 increased (Fig. 3a,b). Furthermore, we cotransfected HuR plasmids and siRNA-HMGB1 (or siRNA-control) into BGC-823 cells and analyzed by CCK-8. We observed that knockdown of HMGB1 inhibited the proliferation induced by HuR overexpression (Fig. 3c). This evidence suggested that elevated HuR in GC cells promoted cell proliferation and was partly mediated by HMGB1.

### Overexpression of HuR contributes to GC cell invasion and migration *in vitro*

HuR plays an important role in cancer invasion and metastasis^8,26^. Studies have revealed that HuR stabilizes several metastasis-related factors, such as matrix metalloproteinase 9 (MMP9), and epithelial-mesenchymal transition (EMT)-promoting factors, such as Snail and ZEB2. To investigate whether HuR overexpression promotes GC metastasis, we analyzed the influence of HuR expression changes on the invasion and migration of GC cells using transwell assays. We observed that knockdown of HuR in MGC-803 cells impaired invasion and migration (Fig. 3d). Meanwhile upregulation of HuR promoted invasion and migration in BGC-823/HuR cancer cells compared to BGC-823/pCMV6 cells (Fig. 3e). Studies also demonstrated that HMGB1 plays an important role in cancer metastasis. Therefore, we tested the influence of exogenous rp-HMGB1 on migration and invasion of BGC-823/HuR cells and MGC-803/si-NC. The results indicated that elevated extracellular HMGB1 level helped to increase migration and invasion ability of cells (Fig. 3d,e).

### Overexpression of HuR promotes GC cell growth *in vivo*

**Figure 4:**
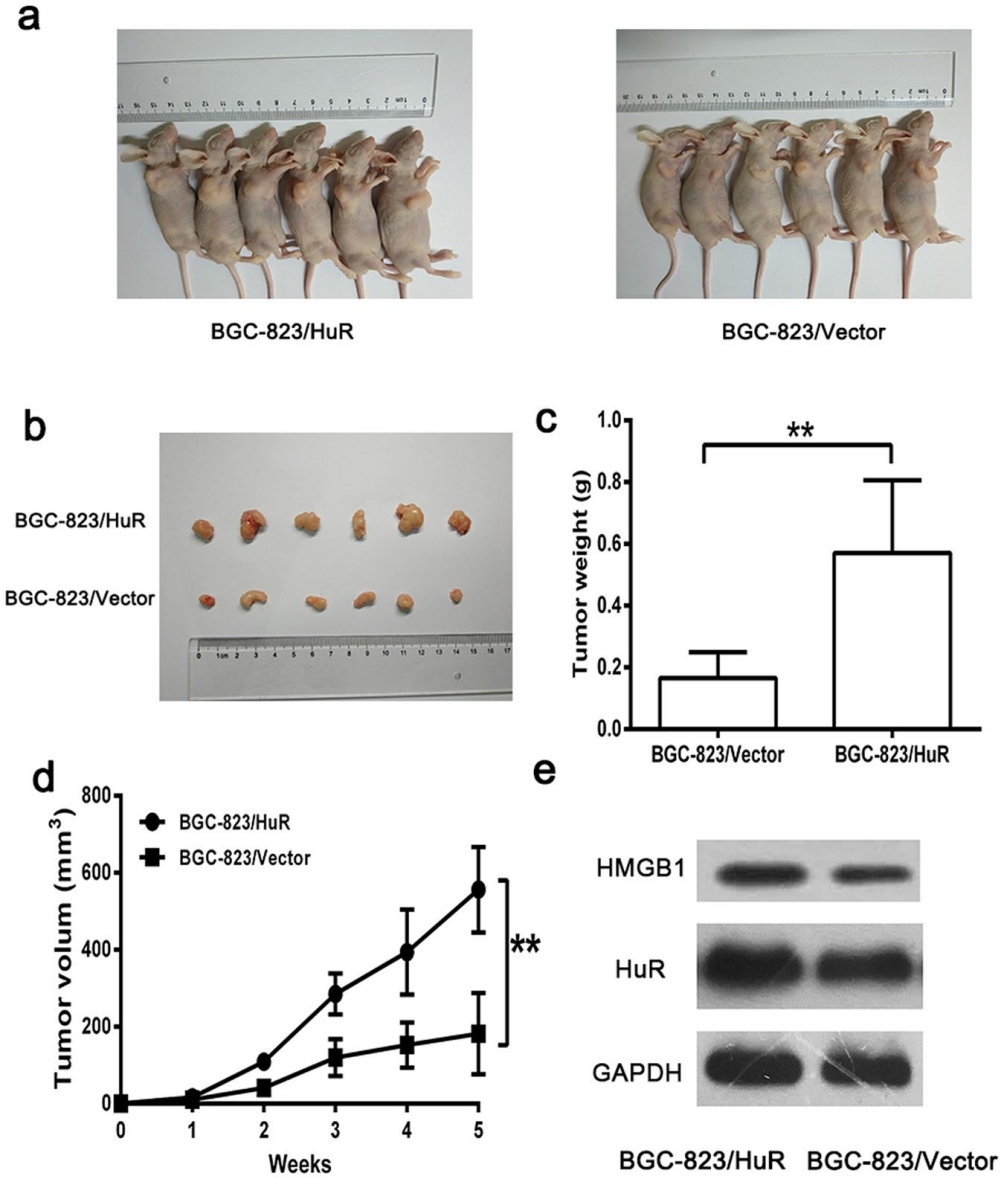
Overexpression of HuR promoted GC cell growth *in vivo*. (**a**,**b**) Representative images of tumors in the two groups after 5 weeks of growth. (**c**,**d**) The tumor volume and weight in BGC-823/HuR group was higher than that in the BGC-823/vector group. (**e**) Elevated expression of HuR and HMGB1 in the subcutaneous tumors was examined by western blotting. The data were represented as the mean ± SD, **p < 0.01.

To investigate whether overexpression of HuR promotes GC growth *in vivo*, we established xenograft tumor models in nude mice. We injected equal numbers of stable HuR-overexpressing BGC-823/HuR cells and BGC-823/vector cells into the subcutaneous tissue of the armpits of nude mice. The size of the xenograft tumors was monitored once per week. Following 5 weeks of growth, the tumors were stripped and measured (Fig. 4a,b). We observed that the average size and weight of the xenograft tumors in models implanted with HuR-overexpressing BGC-823/HuR cells (556.2 ± 45.34 mm^3^; 0.571 ± 0.096 g, respectively) were much higher than those observed in controls (182.0 ± 43.05 mm^3^, *p* < 0.01; 0.166 ± 0.034 g, *p* < 0.01, respectively) after 5 weeks of injection (Fig. 4c,d). Elevated HuR and HMGB1 expression were verified by western boltting (Fig. 4e). These results indicated that HuR overexpression induced significant promotion of GC cell growth *in vivo*.

### The relationship between HuR and clinical features of GC patients

**Figure 5:**
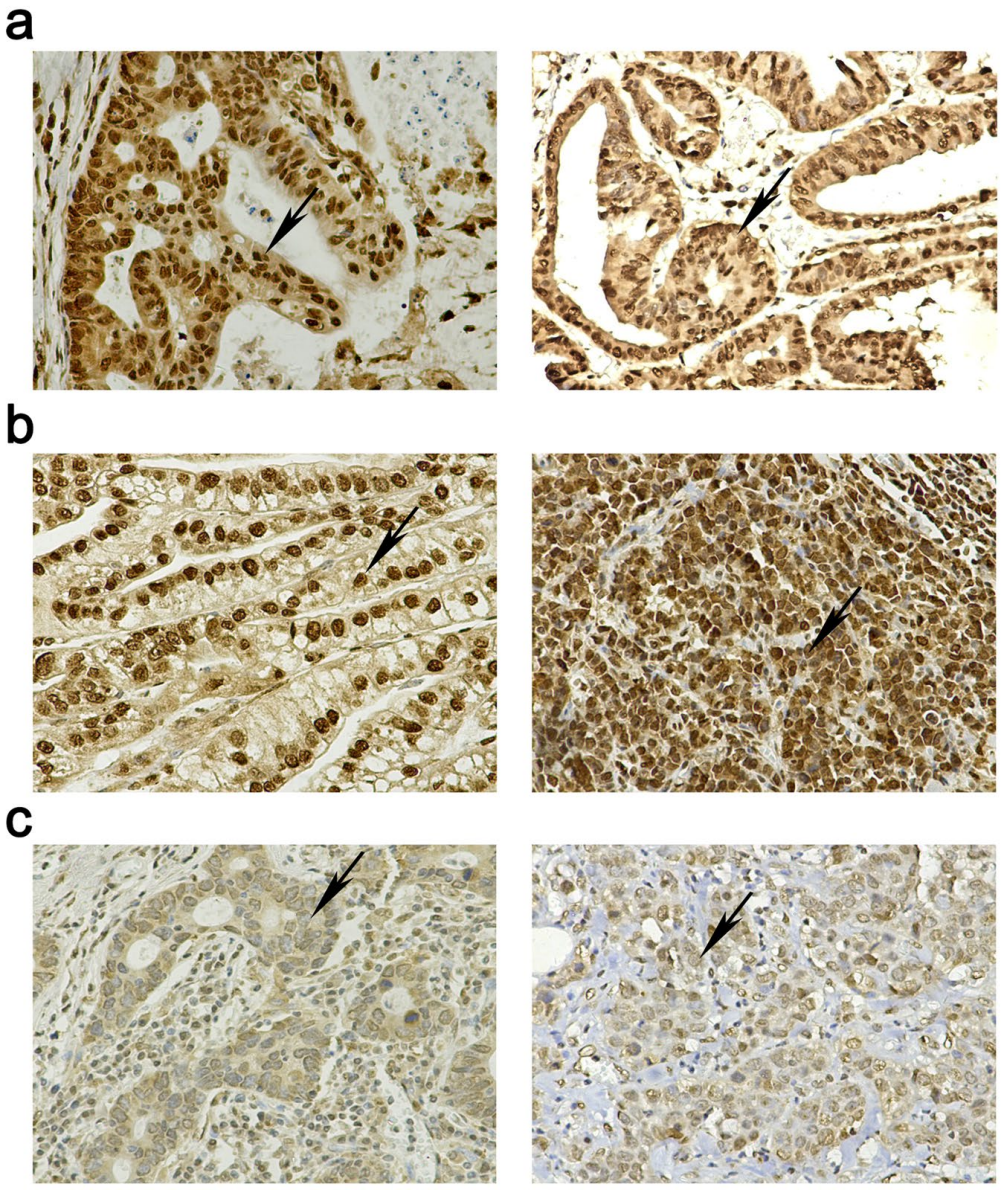
Representative images of HuR immunohistochemical staining in (**a**) the positive cytoplasmic expression group; (**b**) high nuclear expression group; (**c**) low nuclear group (400× magnification).

To further explore the clinical role of HuR in GC, we measured the expression of HuR in 251 cases of GC specimens by immunohistological staining (representative images shown in Fig. 5) and analyzed the correlation between the HuR expression levels and clinicopathological features of GC. Several studies have demonstrated that aberrant cytoplasmic/nuclear distribution of HuR plays an important role in various cancers. Therefore, we analyzed the relationships between cytoplasmic or nucleus HuR expression and pathological features; the results are as shown in Tables 2 and 3. We observed that positive cytoplasmic expression of HuR was closely related to the depth of invasion (*p* = 0.006), lymph node metastasis (*p* = 0.019), distant metastasis (*p* < 0.001) and TNM stage (*p* < 0.001) (Table 2). Using the median IHS score of 6 as a division point, these samples were divided into the high-nuclear HuR group (IHS > 6, n = 166) and low-nuclear HuR group (IHS ≤ 6, n = 85). As summarized in Table 3, statistical analysis demonstrated that high-expression levels of nuclear HuR were correlated with the depth of invasion (*p* = 0.040), TNM stage (*p* = 0.009), tumor size (*p* = 0.019) and TTP expression (*p* = 0.017), but were not correlated with sex, age, distant metastasis, lymph node metastasis, differentiation status or aberrant cytoplasmic HuR expression (Table 3).

**Table 2:** The correlations between cytoplasmic HuR expression and the clinical features of GC patients. *P* value < 0.05 was indicated in bold.

Variable	N	Cytoplasmic HuR expression				*p* value
		negative	%	positive	%	
Sex	251					
Male	178	122	68.5	56	31.5	0.668
Female	73	48	65.8	25	34.2	
Age						
<60	80	60	75	20	25	0.092
≥60	171	110	64.3	61	35.7	
Depth of invasion						
T1-T3	82	65	79.3	17	20.7	**0.006**
T4	169	105	62.1	64	37.9	
Lymph node metastasis						
Absent (N0)	104	79	76.0	25	24.0	**0.019**
Present (N1- N3)	147	91	61.9	56	38.1	
Distant metastasis						
M0	226	163	72.1	63	27.9	**<0.001**
M1	25	7	28.0	18	72.0	
TNM Stage						
1 + 2	116	92	79.3	24	20.7	**<0.001**
3 + 4	135	78	57.8	57	42.2	
Tumor size (cm)						
>5	93	61	65.6	32	34.4	0.578
≤5	158	109	69.0	49	31.0	
Differentiation status						
Well/Moderate	147	101	68.7	46	31.3	0.693
Poor/Undifferentiated	104	69	66.3	35	33.7	
TTP-expression						
TTP-high	101	69	68.3	32	31.7	0.870
TTP-low	150	101	67.3	49	32.7

**Table 3:** The correlations between nuclear HuR expression and the clinical features of GC patients. *P* value < 0.05 was indicated in bold.

Variable	N	Nuclear HuR expression				*P* value
		HuR-high	%	HuR-low	%	
Sex	251					
Male	178	116	65.2	62	34.8	0.613
Female	73	50	68.5	23	31.5	
Age						
<60	80	49	61.2	31	38.8	0.263
≥60	171	117	68.4	54	31.6	
Depth of invasion						
T1-T3	82	47	57.3	35	42.7	**0.040**
T4	169	119	70.4	50	29.6	
Lymph node metastasis						
Absent (N0)	104	63	60.6	41	39.4	0.118
Present (N1-N3)	147	103	70.1	44	29.9	
Distant metastasis						
M0	226	146	64.6	80	35.4	0.123
M1	25	20	80.0	5	20	
TNM Stage						
1 + 2	116	67	57.8	49	42.2	**0.009**
3 + 4	135	99	73.3	36	26.7	
Tumor size (cm)						
>5	93	70	75.3	23	24.7	**0.019**
≤5	158	96	60.8	62	39.2	
Differentiation status						
Well/Moderate	147	95	64.6	52	35.4	0.548
Poor/Undifferentiated	104	71	68.3	33	31.7	
TTP-expression						
TTP-high	101	58	57.4	43	42.6	**0.017**
TTP-low	150	108	72.0	42	28.0	
Cytoplasmic HuR						
Positive	81	58	71.6	23	28.4	0.206
Negative	170	108	63.5	62	36.5	

### Expression level of HuR is correlated with overall survival of GC patients

**Figure 6:**
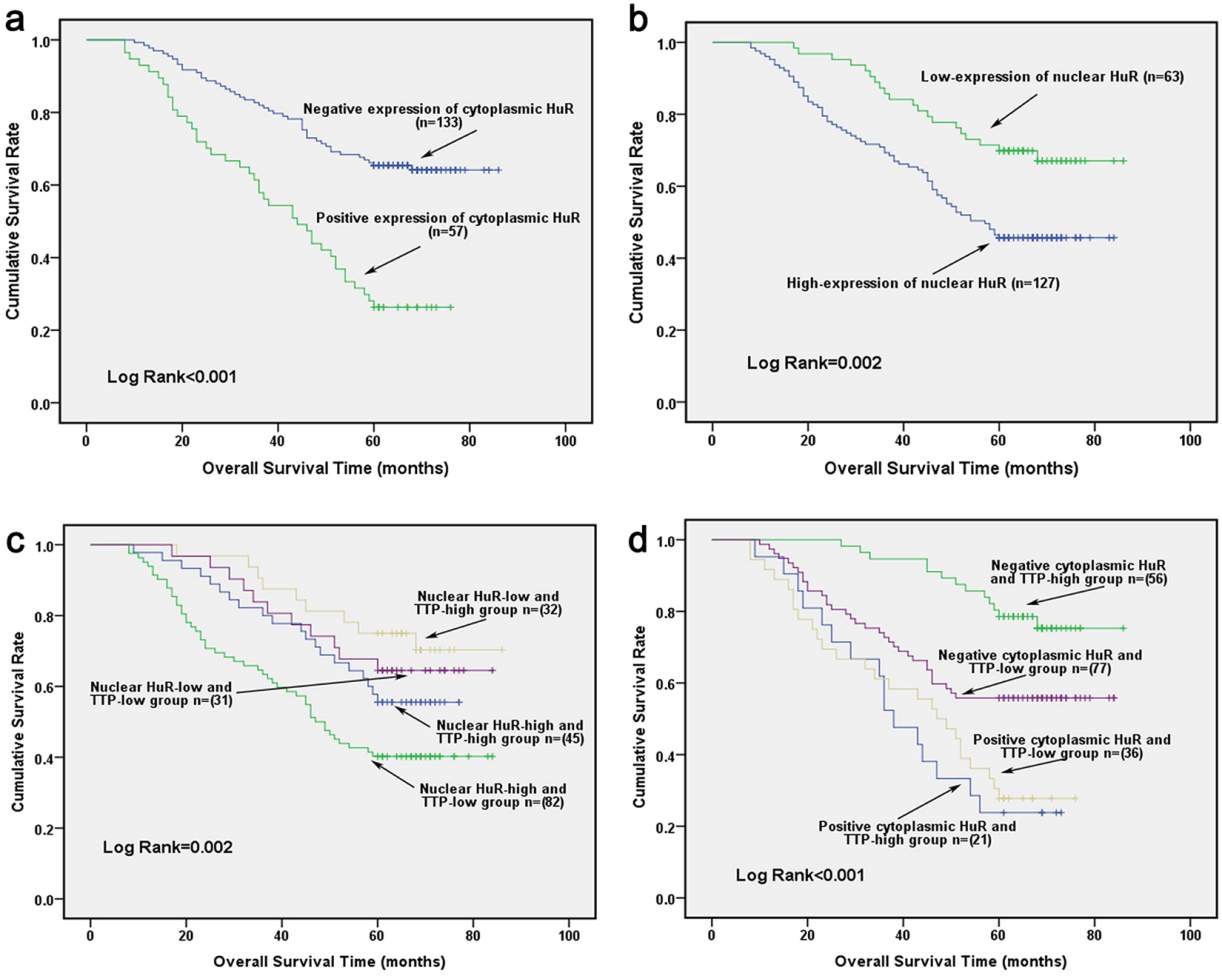
High HuR nuclear expression and aberrant cytoplasmic distribution were associated with poor survival in GC patients. Kaplan-Meier analysis was used to analyze the survival rate in (**a**) different cytoplasmic HuR expression; (**b**) different nuclear HuR expression groups; (**c**) different combinations of nuclear HuR and TTP expression groups; (**d**) different combinations of cytoplasmic HuR and TTP expression groups.

Lastly, to further elucidate whether elevated HuR expression could be used as a prognosis factor in GC, we analyzed the correlation between the overall 5-year survival and HuR expression in 190 cases of GC who received the operations between July 2007 and December 2011 using Kaplan-Meier analysis. As shown in Fig. 6, the results demonstrated that both positive cytoplasmic HuR and high-expression of nuclear HuR always predicted poor survival. The mean survival time in the positive cytoplasmic HuR group was 44.58 months (95% CI: 38.54–50.62), while for the negative cytoplasmic HuR group, it was 68.10 months (95% CI: 63.74–72.46, *p* < 0.001) (Fig. 6a). Furthermore, the mean survival time of patients in the high nuclear HuR expression group was 56.18 months (95% CI: 51.32–61.05), and was evidently shorter than that of the low nuclear HuR expression group (mean survival time: 71.37 months, 95% CI: 65.81–76.93, *p* = 0.002) (Fig. 6b).

In a previous study, we demonstrated that a reduced TTP expression level always predicted poor prognosis in GC patients^16^. As HuR was an important downstream target of TTP, we further analyzed whether the combination of the two factors can be used as a prognostic predictor for the overall 5-year survival of GC patients. As demonstrated, patients with a high nuclear-HuR expression combined with a low-TTP expression pattern had the poorest survival rates (n = 82, 5-year survival rate: 40.2%, mean survival time: 51.951 months, 95% CI: 45.732–58.171), whereas patients with a low nuclear-HuR expression combined with high-TTP expression pattern had the best survival rates (n = 32, 5-year survival rate: 71.9%, mean survival time: 73.625 months, 95% CI: 66.412–80.838) (Fig. 6c). Moreover, we analyzed the survival of patients with different cytoplasmic HuR and TTP expression models. Our results demonstrated that patients with a positive cytoplasmic HuR expression always had poor survival. Furthermore, patients with negative cytoplasmic HuR expression and relatively high TTP levels had the best survival (n = 56, 5-year survival rate: 76.8%, mean survival time: 77.107 months, 95% CI: 72.665–81.549) (Fig. 6d).

To further evaluate whether elevated HuR expression represented a prognostic parameter, Cox’s analysis was applied. Univariate analysis revealed that the depth of invasion, lymph node metastasis, distant metastasis, TNM stage, tumor size, differentiation status, TTP expression, and nuclear and cytoplasmic HuR expression were associated with poor survival rates. Furthermore, Cox’s multivariate analysis revealed that distant metastasis, TNM stage, and both the positive cytoplasmic HuR and relatively high expression of nuclear HuR were independent prognostic factors (Table 4).

**Table 4:** Univariate and multivariate analyses for the overall survival of GC patients. *P* value < 0.05 was indicated in bold.

Variable	*p*-value	Hazard ration (95.0% CI)
**Univariate analysis**		
Age	**0.013**	1.856 (1.137–3.030)
Sex	0.986	0.996 (0.631–1.573)
Depth of invasion (T)	**<0.001**	1.970 (1.416–2.740)
Lymph node metastasis (N)	**<0.001**	1.926 (1.609–2.305)
Distant metastasis (M)	**<0.001**	7.881 (4.717–13.168)
TNM stage (3, 4 vs 1, 2)	**<0.001**	3.306 (2.437–4.485)
TTP expression	**0.018**	0.585 (0.375–0.910)
Nuclear HuR expression	**0.003**	2.137 (1.298–3.517)
Tumor size	**<0.001**	2.832 (1.859–4.316)
Differentiation status	**0.042**	0.649 (0.428–0.984)
Cytoplasmic HuR expression	**<0.001**	3.172 (2.136–4.710)
**Multivariate analysis**		
Cytoplasmic HuR expression	**0.020**	1.699 (1.088–2.654)
Nuclear HuR expression	**0.031**	1.748 (1.054–2.900)
Distant metastasis	**0.001**	3.432 (1.971–5.977)
TNM stage (3, 4 vs 1, 2)	**0.010**	3.057 (1.050–6.471)

## Discussion

Post-transcriptional regulation plays an important role in orchestrating the fates of RNAs. The progression of mRNAs through splicing, maturation, transportation, localization, and degradation is tightly controlled by post-transcriptional regulatory factors, including miRNAs, long non-coding RNAs, RNA-binding proteins and other factors^27^. Transcripts of a lot of cytokines, chemokines, growth factors, and so on are highly enriched of AREs, which interact with ARE-BPs to regulate the stability and translation of these mRNAs^28^. Mutations in binding regions, dysregulation of ARE-BPs expression, aberrant interplay networks with miRNAs and other factors may alter the interaction between ARE-BPs and ARE-containing transcripts and result in elevated expressions of tumor-promoting targets^27^.

Aberrant expression and the function of ARE-BPs have been reported in several types of cancers. Both TTP and HuR are two closely related ARE-BPs and are always dysregulated in cancers^3^. TTP is an mRNA-destabilizing factor and acts as a tumor suppressor, while HuR stabilizes target mRNAs and helps promote the translation of some mRNAs. Expression of TTP is decreased, while HuR is overexpressed or aberrantly distributed in the cytoplasm in several types of cancers. Interestingly, both TTP and HuR are highly subject to extensive posttranslational modifications, especially phosphorylation. Various protein kinase, for example p38 MAPK, ERK/MAPK, and MK2 are associated with their phosphorylation^5^. Phosphorylated TTP interacts with the multifunctional 14-3-3 adaptor proteins to form complexes, which protect TTP proteins from degradation and inhibit TTP mediated target mRNA decay^29^. As for HuR, modifications including phosphorylation and methylation at different residues affect its subcellular localization and interaction with target mRNAs^14^. Moreover, TTP downregulates HuR mRNA levels^11^. According to a recent study, thousands of overlapping binding sites of TTP and HuR were found in more than one thousand genes, and most of the TTP sites in the 3′UTRs overlapped with HuR binding sites^13^. Despite both TTP and HuR are ARE-BPs, their binding sites are not completely equivalent. TTP prefers to bind to AU rich elements and its optimum binding sequence is the AUUUA motif flanked by additional uridylate residues, whereas HuR can bind to the AU-, CU- and U-rich elements of target mRNAs.

Dysregulation of the TTP-HuR axis may increase a series of pro-tumorigenesis factors that are associated with the development and progression of several of cancers, which we have previously reviewed^3^. An aberrant TTP-HuR axis is associated with proliferation, apoptosis, angiogenesis, metastasis, invasion, and resistance to chemotherapy. For example, elevated HuR in GC facilitated DNA synthesis, the G_1_ to S phase transition of cell cycle and acted as an anti-apoptotic factor^30^. And HuR interacts with the 3′ UTR of matrix metalloproteinase 9 (MMP-9), urokinase plasminogen activator (uPA) and its cell surface receptor uPAR to increase cancer invasion ability. TTP is a regulator of MMP-1, -2, -9, uPA/uPAR and other factors which play important roles in tumor metastasis^3,11^. Our previous work has demonstrated that TTP is downregulated in GC. Decreased expression of TTP is closely related to the depth of invasion, TNM stage, lymph node metastasis and survival in GC patients. Restoring TTP expression in GC cells inhibits the proliferation, invasion and migration of cancer cells^16^. In this study, elevated expression of HuR and aberrant cytoplasmic translocation were observed in GC. Using immunohistochemistry staining, we found that both positive cytoplasmic localization and elevated nuclear HuR expression were closely related to poor clinicopathological features and survival of GC patients.

Furthermore, we observed that dysregulation of TTP and HuR induced elevated expression of HMGB1 in different ways. Increasing evidence indicates that HMGB1 is a multifunctional factor with diverse biological functions that depend on the context, modification and its location. As a non-histone chromosomal protein in the nucleus, HMGB1 participates in DNA replication, repair, and transcription. Elevated HMGB1 in the cytoplasm and extracellular environment is associated with a variety of activities, including inflammation, autophagy, anti-apoptosis, proliferation, metastasis, among others^31^. HMGB1 plays a paradoxical role in cancers. It acts as both a cancer-promoter and a tumor-suppressor^19^. For example, acting as an anti-tumor factor, intracellular HMGB1 sustains autophagy and stabilizes the genome. Overexpressed HMGB1 in breast cancer was reported to bind to the tumor suppressor Rb and induce cell cycle-arrest and apoptosis^32^. However, extracellular HMGB1 interacts with different receptors, including RAGE, TLR-2, and TLR-4, and acts as a cytokine, chemokine, and growth factor to promote carcinogenesis^33^. Elevated HMGB1 levels are associated with poor survival of cancer patients^23,34^. Several studies have certified that HMGB1 is overexpressed in GC^17,19,24,35^. And higher serum HMGB1 levels have also been reported in GC patients compared to those in normal patients^24^.

However, the mechanism by which HMGB1 is overexpressed in cancers is not fully elucidated^20^. A recent study reported that HuR bound to U-rich elements in the 3′UTR of HMGB1 and promoted its translation in the progression of myogenesis. In accordance with this report, we observed that elevated HuR had no effects on mRNA expression of HMGB1, but promoted its expression at translational level. In this study, we observed that downregulated TTP-expression increased HMGB1 expression at mRNA level by binding to its ARE directly. Dysregulation of the TTP and HuR in GC promoted the proliferation and metastasis of GC cells in part by elevated levels of HMGB1. Soluble RAGE, the HMGB1 antibody, and other agents, such as glycyrrhizin and quercetin that target HMGB1 showed a promising prospect for anti-cancer therapy^20^. Thus, rebalancing the TTP-HuR axis showed potency in reducing cancer. For example, inhibition of miR-29a, a miRNA that recognizes a seed target site on the 3′UTR of TTP, rebalanced the TTP-HuR axis and reduced the invasiveness of breast cancer cells^11^.

Collectively, our results demonstrated that dysregulation of TTP and HuR played an important role in GC. The increased HuR levels were partly due to the downregulation of TTP. Positive cytoplasmic HuR expression was closely related to the depth of invasion, lymph node metastasis, distant metastasis and TNM stage of GC patients. Elevated nuclear HuR levels were correlated with the depth of invasion, TNM stage, tumor size and TTP expression in GC patients. Aberrant cytoplasmic HuR and elevated nuclear HuR levels predicted a poor survival in GC patients. Additionally, we demonstrated that elevated expression of HMGB1 was correlated with dysregulation of TTP and HuR. Furthermore, different mechanisms of regulating HMGB1 expression by TTP and HuR were observed in GC. TTP regulated the expression of HMGB1 at the mRNA level by binding to its ARE, while elevated HuR mainly promoted its expression at translational level. Elevated HuR expression promoted proliferation, invasion and migration of GC cells partly through HMGB1. Increased HuR levels in a xenograft tumor model promoted tumor growth of GC cells *in vivo*. The underlying mechanisms by which the dysregulation of TTP and HuR promotes cancer progression are worthy for further research. And the TTP-HuR axis might serve as a potential therapeutic target and prognostic indicator for GC patients.

## Materials and Methods

### Ethics statement

This study was approved by the ethics committee of Nanjing medical university and the written informed consents were obtained from all participated patients. All experiments with mice were approved by the Animal Care and Use Committee of Nanjing Medical University. All experimental methods involving both human participants and mice were performed in accordance with the approved guidelines by the ethics committee of Nanjing medical university and the Animal Care and Use Committee of Nanjing Medical University.

### Cells and patients’ specimens

Gastric cancer cells (MGC-803, BGC-823, SGC-7901, MKN-45) and the human gastric epithelial GES-1 cells were stored and maintained in the central laboratory of wuxi second peoples’ hospital. A total of 70 pairs of fresh GC specimens and related adjacent tissues were collected for RNA extraction or immunohistochemistry from GC patients who underwent the gastrectomy at the general surgery department of wuxi second peoples’ hospital from October 2012 to January 2015. Besides, 251 resected paraffin-embedded gastric cancers samples who received radical operations with standard D_2_ or extended lymph node dissection were also collected for immunohistochemistry (including 190 cases who received the operations between July 2007 and December 2011 and their survival data were collected for further survival analysis).

### RNA preparation, reverse transcription-polymerase chain reaction (RT-PCR) and quantitative real-time PCR (qRT-PCR)

Total RNA was extracted by TRIzol Reagent (Invitrogen, CA, USA) from cells or fresh samples according to the manufacturer’s protocol. Next, RT-PCR was performed with 1 µg of total RNA using a PrimeScript RT Reagent Kit with a gDNA eraser (Takara, Dalian, China). Target mRNA expression was determined by qRT-PCR using the QuantiFast SYBR Green PCR Kit (Qiagen, Germany) on an ABI Step One Plos Fast real-time PCR system (Applied Biosystems, Austin, USA). The primer sequences used in this study were as follows: TTP-forward, 5′-TTCGCCCACTGCAACCTC-3′; TTP-reverse, 5′-CGCCCACTCTCTGAGAAGGTC-3′; HuR-forward, 5′-CCGTCACCAATGTGAAAGTG-3′; HuR-reverse, 5′-TCGCGGCTTCTTCATAGTTT-3′; HMGB1-forward, 5′-TATGGCAAAAGCGGACAAGG-3′; HMGB1-reverse, 5′-CTTCGCAACATCACCAATGGA-3′; Glyceraldehyde-3-phosphate dehydrogenase (GAPDH)-forward, 5′-AAGGTGAAGGTCGGAGTCAA-3′; GAPDH-reverse, 5′-AATGAAGGGGTCATTGATGG-3′. The specificity of primers was verified by the melting curve and agarose gel electrophoresis. The results of target mRNA expression were normalized to GAPDH and quantified by the 2^−ΔΔCT^ method. Transcription of mRNAs was blocked by actinomycin D (ActD) (5 µg/ml) to estimate the stability of target mRNAs. All samples were measured at least in triplicate.

### DNA transfection and RNA interference

The pcDNA-TTP plasmid and pcDNA 3.1 empty vector were constructed as previously described^16^. The pCMV6-HuR (RC201562) expression plasmids as well as the pCMV6-Entry (PS100001) empty vector were purchased from OriGene (OriGene Technologies, Beijing, China). Small interfering RNAs (siRNA) of TTP, HuR, HMGB1 and the control siRNA (si-NC) were purchased from Riobo Bio (Riobo Bio, Guangzhou, China). Plasmids and siRNAs were transfected into cells cultured on 6-well plates using Lipofectamine2000 (Life Technologies, USA). Next, total RNA and protein were harvested after 24 h or 48 h for further analysis. The efficiency of the plasmids and siRNAs were verified by either qRT-PCR or western blotting.

### Luciferase assay

The wild type sequence of HMGB1 3′UTR (wtARE) and the mutant oligonucleotides in which all of the 14 ATTTA motifs were substituted with AGGTA (mutARE) were synthesized and subcloned into the pmirGLO Vector by Gene Pharma company (Suzhou, China). The 293 T cells were cotransfected with pmirGLO-wtARE or pmirGLO-mutARE vectors (0.5 µg) and TTP plasmids (0.5 µg). The lysates of the transfected cells were mixed with luciferase assay reagent in accordance with the manufacturer’s instructions (Promega, USA), then the chemiluminescence was measured using a Wallac Vector 1420 multi-label counter (EG&G Wallac). The *renilla* luciferase activities of the pmirGLO-wtARE/mutARE were used to normalize the *firefly* luciferase activities.

### Protein extraction and western blotting

Total protein of cells was extracted using the cell lysis buffer (9806, Cell Signaling Technology, USA) supplemented with phenylmethylsulphonyl fluoride (PMSF) (1 mM, final concentration). Nuclear and cytoplasmic proteins were extracted using a nuclear and cytoplasmic protein extraction kit (P0027, Beyotime, China). Next, the concentrations of extracted proteins were measured by enhanced BCA protein assay kit (P0010S, Beyotime, China). Briefly, equal amounts of proteins were loaded and separated by 12% sodium dodecyl sulfate-polyacrylamide gel electrophoresis (SDS-PAGE). After the proteins were transferred onto polyvinylidene difluoride (PVDF) membranes and blocked in 5% non-fat milk, they were incubated with appropriate dilutions of primary antibodies at 4 °C overnight. The signals were detected by Immobilon^TM^ Western Chemiluminescent HRP Substrate (Millipore, USA). Lamin B1 was used as loading control for nuclear proteins. The primary antibodies were as follows: tristetraprolin (ab33058, Abcam), HuR (11910-1-AP, Proteintech, China), HMGB1 (10829-1-AP, Proteintech, China), β-actin (60008-1-Ig, Proteintech, China), GAPDH (60004-1-Ig, Proteintech, China), Lamin B1 (12987-1-AP, Proteintech, China).

### Immunohistochemistry

Immunohistochemistry was performed on 4-µm-thick sections obtained from formalin-fixed and paraffin-embedded tissue blocks. Sections were incubated overnight with antibodies against TTP (1:100) and HuR (1:100) after antigen retrieval using sodium citrated buffer and inactivation of endogenous peroxidases by 0.03% hydrogen peroxide as well as normal goat serum (Solarbio, China) blocking. Finally, immunohistochemical staining was conducted using a MaxVision HRP-Polymer anti-rabbit IHC Kit and diaminobenzidine (DAB) kit (Maixin Biotech, China). Slides subsequently were counterstained with hematoxylin. Negative immunohistochemistry controls were established using PBS instead of primary antibodies.

Two independent and experienced pathologists were invited to assess the expression of target proteins. The level of target protein expression was semi-quantitatively estimated by an immunostaining score (IHS) that was the product of the intensity of the staining score and percentage of positive cells. The intensity of staining was scored as follows: 0, negative staining; 1, weak staining; 2, moderate staining; 3, strong staining. The percentage of positive cells was scored as follows: 1, (0–25% positive cells); 2, (26–50% positive cells); 3, (51–75% positive cells); 4, (76–100% positive cells). Each patient was classified into the low-expression or high-expression group based on the IHS (0–6, low-expression group; 8–12, high-expression group).

### Cell proliferation assay

Cell proliferation was analyzed using the Cell Counting Kit-8 (CCK-8) reagent (Dojindo Laboratories, Kumamoto, Japan). Approximately 2 × 10^3^ cells were seeded into 96-well plates. Then CCK-8 reagents were added to each well according to manufacturers’ protocol. Next, the absorbance of each well was measured at 450 nm by Multiskan GO (Thermo Fisher Scientific) to assess cell counts.

### Cell migration and invasion assays

We used CoStar Transwell chambers (8-μm pore size, Corning, NY, USA) to analyze the migration ability of GC cells and used chambers which were coated with Matrigel (BD Biosciences) to assess the invasion ability. Cells (1 × 10^5^/well) in serum free medium were plated into the upper chambers of the wells and the bottom chambers were placed in the medium with 10% FBS. After incubation at 37 °C in 5% CO_2_ for an appropriate time, we removed the remaining cells at the upper surface of the membrane. Next, cells on the lower side of the membrane were fixed with 4% paraformaldehyde and stained with crystal violet. The mean stained cells numbers of five random fields (at ×200 magnification) in an optical microscope were calculated to assess the migration and invasion ability. Recombinant HMGB1 protein (100 ng/ml) (Z02803-1, Genscript Corporation, USA) was used to assess the role of HMGB1 in cancer cell proliferation and metastasis potential.

### ***In vivo*** experiments

All experiments with mice were approved by the Animal Care and Use Committee of Nanjing Medical University. BGC-823 GC cells (relatively low HuR expressers) were transfected with the pCMV6-HuR and pCMV6-Entry plasmids and then selected with G418 (500 µg/ml) at 48 hours of post-transfection. Expression of HuR was confirmed by western blotting, and positive clones were picked for further expansion. Twelve eight-week-old BALB/c nu/nu mice were purchased from the Experimental Animal Center of Yangzhou University and randomly divided into two groups (six mice in each group). Both groups received subcutaneous injections of either HuR-overexpressing or BGC-823/vector cells (n = 5 × 10^6^ cells in 200 µl of PBS). The generated tumor volumes were measured with a digital caliper and calculated by the modified ellipsoidal formula: (length × width^2^)/2. After 5 weeks of observation, nude mice were sacrificed and tumors were harvested and then frozen in liquid nitrogen for further protein and RNA extraction.

### Statistical analysis

Statistical Package for Social Science (SPSS) 20.0 (SPSS Inc, USA) was used for all statistical analyses. Difference between groups was analyzed by one-way analysis of variance (ANOVA) or Student’s t-test. Correlations between HuR protein expression and the clinical pathological features were assessed by the Chi-square test. Kaplan-Meier analysis was used to calculate survival, and the significance of these curves was evaluated using the Mantel–Cox log-rank test. *P* value < 0.05 was considered statistically significant.

## Electronic supplementary material

Supplementary information
