# Supplementary material for: Dysregulation of tristetraprolin and human antigen R promotes gastric cancer progressions partly by upregulation of the high-mobility group box 1
Source: Sci Rep. 2018 May 4;8:7080. doi: 10.1038/s41598-018-25443-3 (PMC5935726; doi:10.1038/s41598-018-25443-3)
Supplement: Supplementary file 1 — Supplementary information [file 41598_2018_25443_MOESM1_ESM.docx]

**Supplementary Information**

**Dysregulation of tristetraprolin and human antigen R promotes gastric cancer progressions partly by upregulation of the high-mobility group box 1**

Hao Wang1, Yigang Chen1, Jian Guo1, Ting Shan1, Kaiyuan Deng1, Jialin Chen1, Liping Cai1, Hong Zhou1, Qin Zhao1, Shimao Jin1, Jiazeng Xia1*

1 Department of General Surgery and Translational Medicine Center, Nanjing Medical University Affiliated Wuxi Second Hospital, Wuxi 214002, China.

Correspondence to: Dr. Jiazeng Xia*, email: jiazengxia@yahoo.com


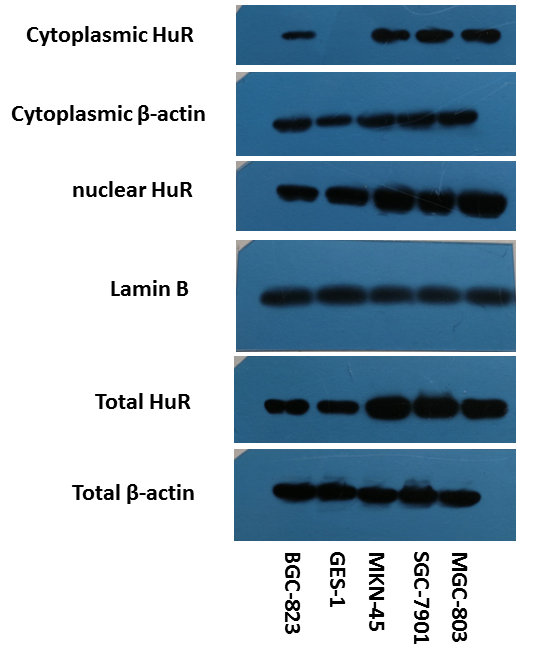


Supplementary Figure S1. Full-length western blots related to Figure 1. Expression of HuR was elevated in GC. b. Positive cytoplasmic HuR expression was detected in MGC-803, SGC-7901, MKN-45, and BGC-823 cells, whereas no cytoplasmic expression of HuR was found in GES-1 cells.


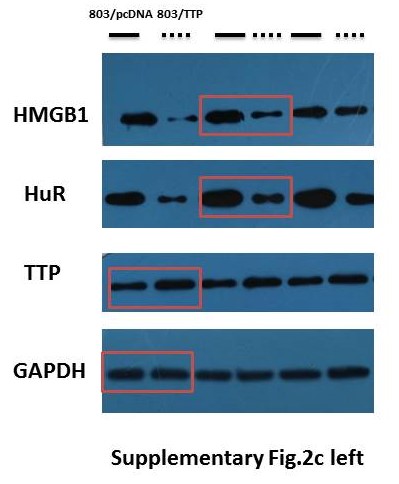

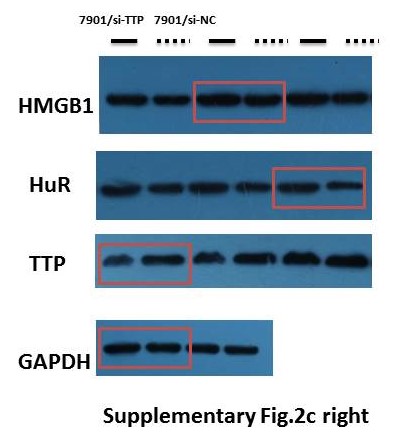

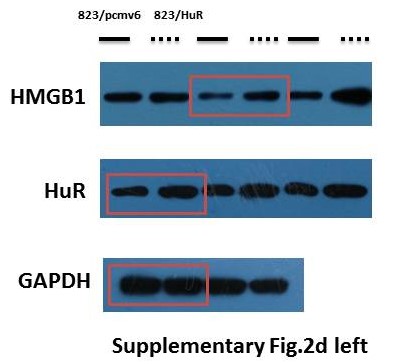


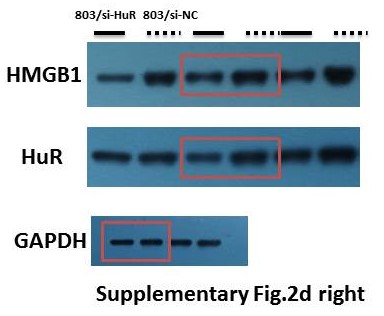


Supplementary Figure S2. Full-length western blots related to Figure 2. Western blotting analysis demonstrated that: (c) TTP regulated the expression of HuR and HMGB1. (d) HuR upregulated the expression of HMGB1 at the translational level. Bands shown in Figure 2 are marked with red boxes.


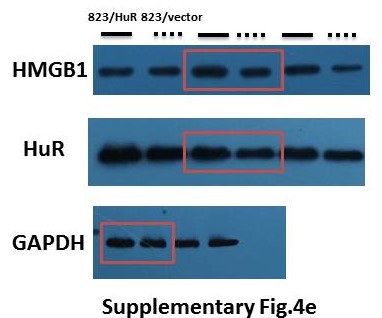


Supplementary Figure S4e: Full-length western blots related to Figure 4e. Overexpression of HuR promoted GC cell growth *in vivo.* (e) Elevated expression of HuR and HMGB1 in the subcutaneous tumors was examined by western blotting. Bands shown in Figure 4e are marked with red boxes.

**western blotting**

For western blotting, we firstly extracted the total protein of cells measured by enhanced BCA protein assay kit. Briefly, equal amounts of proteins were loaded and separated by 12% sodium dodecyl sulfate-polyacrylamide gel electrophoresis (SDS-PAGE) and further verified by the internal reference protein (GAPDH, β-actin and Lamin B1). Because the observed antibodies molecular weights were approximate to each other (tristetraprolin (36 kDa), HuR (30-37 kDa,), HMGB1 (30 kDa), β-actin (42 kDa), GAPDH (36 kDa)), we subsequently reloaded the equal amounts of proteins and separated by SDS-PAGE and transferred onto PVDF membranes. After blocked in 5% non-fat milk, they were incubated with appropriate dilutions of primary antibodies at 4 °C overnight. The signals were detected by Immobilon^TM^ Western Chemiluminescent HRP Substrate (Millipore, USA).
